# Pragmatic Profiles of Toddlers With Autism Spectrum Disorder at the Onset of Speech

**DOI:** 10.3389/fneur.2020.612314

**Published:** 2021-01-18

**Authors:** Alona Oren, Esther Dromi, Sheila Goldberg, Aviva Mimouni-Bloch

**Affiliations:** ^1^The Pediatric Neurology and Developmental Unit, Loewenstein Rehabilitation Medical Center, Raanana, Israel; ^2^Constantiner School of Education, Tel Aviv University, Tel Aviv, Israel; ^3^Sackler Faculty of Medicine, Tel Aviv University, Tel Aviv, Israel

**Keywords:** autism spectrum disorder, toddler (MeSH), early language, development, pragmatics, communicative intention, naturalistic interaction

## Abstract

Using speech to communicate pragmatic functions is challenging among individuals with Autism Spectrum Disorder (ASD). Given the role language plays in developing everyday skills, we traced the unique pragmatic profile of early words, seeking comparison to typically developing (TD) toddlers at similar lexical stages. Twenty-four mother-toddler dyads participated (9 ASD and 15 TD). Dyads were video recorded when toddlers reached a productive lexicon of 40–70 words. These recordings were captured three times during naturalistic interaction and at two consecutive visits with a 2-month interval. Seven thousand three hundred seventy-six productions were analyzed and classified into four communicative intentions (Declaratives, Requests, Objections, and Non-Communicative speech). ASD toddlers were delayed in the emergence of words compared to TD toddlers, with a greater within-group variability (median 28 months, IQR 24.5–35, median 17 months, IQR 17–18, respectively, *p* < 0.001). In both groups, the most common communicative intention was Declarative. However, the percentage of Declaratives was higher among TD toddlers across visits compared to ASD toddlers. In both groups, most productions were directed toward the communicative partner, but ASD toddlers used Non-Communicative speech more often than TD peers. Non-Communicative speech gradually decreased over time. We conclude that while TD toddlers begin to talk with an already-established knowledge of the main communicative functions of words, ASD toddlers seem to have only a partial understanding and gradually improve communicative use as they expand their lexicon. These findings bear theoretical and practical implications for early intervention in ASD. We suggest that communicative profiles are affected by individual characteristics and by the interaction style.

## Introduction

Language learning in young children with Autism Spectrum Disorder (ASD) is characterized by great variability (1, 2). Quite often, toddlers with ASD exhibit delays in the emergence of first words in comparison to age-mates with typical development (TD) (3–5). Both differences and similarities have been reported between ASD children and language-matched TD children, regarding word composition profiles (6), the application of various mechanisms for language learning (7–10), and the association between language production and development in other domains (11). Therefore, the question of whether ASD toddlers follow the typical path to acquiring a language cannot be answered unequivocally. However, there seems to be an agreement that the use of language to communicate in a manner sensitive to the context, otherwise known as pragmatics, is impaired throughout the Autistic Spectrum. Such pragmatic deficits are present even among highly verbal or high functioning individuals with ASD (11, 12). Research on pragmatic deficits focuses either on nonverbal communicative skills of pre-verbal toddlers with autism (e.g., pointing) (13) or on higher-level pragmatic abilities such as carrying conversation (14). Research on the way ASD toddlers use their early words to communicate is still lacking.

The transition from pre-verbal communication to spoken language seems to be impaired in ASD children. TD infants use gestures and vocalizations a few months before their first birthday to convey basic communicative intentions such as simple requests, protests, and declaratives. These nonverbal means are prerequisites for the learning of shared, conventional meanings of words (15). As words appear, they serve similar communicative functions to those represented by the nonverbal means and address similar referents (16). Of note, the typical transition from pre-verbal communication to speech does not occur in toddlers with ASD who demonstrate restricted use of gestures and pre-verbal productions (17, 18). The matter of how ASD toddlers use their first words for conveying their communicative intentions has yet to be explored.

As the essence of pragmatics is using speech in context, we suggest that communicative intentions should be assessed within the setting of naturalistic interactions. It is agreed that a familiar context and a familiar partner have an optimal influence on the toddler's communicative functioning; thus, we believe that direct observations of dyads' naturalistic interactions are a vital tool for corroborating findings from questionnaires (19, 20).

When attempting to predict which communicative intentions are expressed via early words, it seems reasonable to assume a developmental course similar to that found in nonverbal communication. Several studies referred to the referential deficit, stating that gestures serving requests and protests (e.g., reaching) are relatively similar in the ASD population to those of the TD. On the other hand, lack of declarative (e.g., showing objects), is one of the core features observed in toddlers with ASD (13, 21, 22). If one assumes a continuity between pre-verbal and verbal means for communication, it would be reasonable to expect that the majority of early words would convey requests rather than declaratives. In addition, taking into account that difficulties in cooperation and sustaining interaction are common in ASD, one could also assume that the proportion of words used for protesting would be greater among toddlers with ASD than among TD toddlers at a similar lexical level. Last, given the well-documented tendency for speech for the self, we assumed that this proportion of “Non-Communicative Speech” would be greater in toddlers with ASD than in TD toddlers at similar lexical levels (23). The goal of the present study was to classify the communicative intentions that toddlers with ASD express at the onset of speech and to compare the distribution of communicative intents in this group to that of TD toddlers at similar lexical levels. In addition, we examined the trajectory of early words learning in the two groups.

## Methods

### Participants

All participants came from monolingual Hebrew-speaking typical families (one mother and one father). ASD toddlers were recruited via advertisements in developmental centers and early intervention programs. Toddlers received a diagnosis of ASD (DSM-5) by an experienced multidisciplinary clinical team in one of the public developmental centers in Israel. The diagnosis of ASD was confirmed using the Autism Diagnostic Observation Schedule (17). TD toddlers were recruited by advertisements in social media. Children diagnosed with genetic and chronic medical conditions were excluded from both groups. Normal hearing status was confirmed for all participants.

To exclude minimally verbal toddlers, we set an age limit of 48 months for ASD participants. A limit of 24 months was set to exclude language delays in the TD control group.

Of 36 dyads initially screened, 12 were excluded due to medical conditions, being bilingual, having a non-typical family structure, having a vocabulary exceeding the criterion or, in the case of TD toddlers, having any form of atypical development.

The final ASD sample included nine toddlers (seven boys, two girls).

Cognition was evaluated with either Bayley or Mullen examination. Six children scored extremely low IQ and were defined by DSM 5 (18) as requiring very substantial support. The other three children needed substantial support while one of them scored low average IQ and two scored average. During the period of the study all nine ASD participants were enrolled in rehabilitation daycare centers. Treatment typically consisted of 12–14 weekly hours of therapy including parental guidance, speech, occupational, and psychologic therapy in an “eclectic” approach (24, 25). The final TD sample included 15 toddlers (13 boys, two girls). A comparison between the two groups showed no differences in parental educational level or socio-demographic background variables.

### Tools and Measurements

#### A Playing Kit

To create a unified context as a base to comparison of the free play environment, we provided a kit of toys. The kit included age-appropriate toys such as wooden puzzles, pop-up puppets, bubbles, balloons, a book, and a doll house which typically provide opportunity for mutual play and function as communicative temptations (26–28).

#### Hebrew Communicative Development Inventory- Words and Gestures (HCDI- WG)

Each child's expressive level was evaluated using the Hebrew standardized version of the McArthur-Bates Communicative Development Inventory (MBCDI-WG). This report consists of lists of early gestures and words, each classified to either “understands” or “produces.” This tool demonstrated high reliability in all questionnaire components with alpha Cronbach ranging from α = 0.65 to α = 0.98 (29).

#### Classification of Early Words Into Communicative Intentions

All verbal productions of each child during dyadic interaction with his/her mother constituted the data set for the present study's analysis. Productions were analyzed providing they were either sound effects (e.g., “MEOW” for cat) or any verbalization that consisted of at least a single syllable (e.g., “BA” for “Bu-bah” = a doll). Crying and shouting were excluded from the word production analysis.

Given the lexical stage, we set the code for classifying communicative intents to address four exclusive categories: Requests, Declaratives, Objections, and Non-Communicative productions, and a fifth “other” default category. Table 1 presents the operational definitions for each communicative intention.

**Table 1:** Definitions for pragmatic categories.

**Communicative intention**	**Definition**	**Example**
Request	A verbal production addressed to the partner in order to receive a desired object or to perform an action	Child hands the mother a closed box, saying “open!”
Declarative	A verbal production intended to share knowledge or to get the partner's approval/ attention	Child points at a picture of a cow and says “Moooo”
Objection	A verbal production intended to stop or prevent an undesired event	Child says “no” while pushing the object or nodding upon being offered an object
Non-communicative speech	A verbal production that does not appear to be addressed to a communicative partner, may serve as self-stimulatory or practice	Child appears distant or disconnected, makes no eye contact, and recites a poem.
Others	A verbal production that was not necessarily addressed to another person or its communicative intent is unclear.	The child makes an unintelligible verbal production (“ARGH”) and it cannot be determined whether it is a communicative act

#### Interact ® Software

In order to perform the pragmatic analysis of intentions, we utilized a computerized software (INTERACT ® software 14th edition), which enables frame by frame encoding of simultaneous verbal and nonverbal behaviors of the toddlers and their mothers. A timeline of behaviors was determined within the highly compressed interaction, making it possible to track changes in both form and frequency of each verbal production, including its antecedent and subsequent behaviors by both partners in the interactions.

#### Procedure

The study was approved by the local hospital and the university Institutional Review Boards. Parents signed an informed consent form. Direct observations in the homes of each child took place on three occasions with a 2-months' interval between any two visits. Shortly prior to entering the study, mothers tracked the productive lexicon of their child using the HCDI-WG questionnaire. The first visit was scheduled when the toddler's expressive lexicon reached 40–70 different words. The questionnaire was refilled in proximity to the second and third visits, thus reflecting the accumulating lexicon of the child. In each visit, dyads were video recorded during a 30-min free play session. Mothers were instructed to play with their child as they would normally while using the toy kit as much as possible. A sample of the first uninterrupted 15 min of each play session was coded. Each toddler's verbal production was transcribed alongside with the nonverbal behaviors that accompanied it using the INTERACT software. A timeline of behaviors was formed, thus enabling the analysis of contingencies and other relationships among the toddler's verbal productions and non-linguistic and linguistic behaviors. The present study focused on verbal productions.

#### Judging Communicative Intentions and Reliability Measures

Since parents are experienced in judging communicative intentions conveyed by toddlers' verbal productions, we recruited a mother to conduct the pragmatic analysis of the video recordings. To keep judgment as intuitive as possible, the mother-judge was introduced to the main communicative categories and was assured that the manifestation of each intention may vary in form and appear in various contexts. She was asked to watch two recordings along with the first author and to raise her concerns or questions. In order to examine the reliability, another mother with similar demographic characteristics was similarly trained using the same protocol.

Reliability regarding the pragmatic intentions of word productions was carried out by the two independent judges who coded 12% of the complete data set. The samples were derived from nine different participants from both groups and different visits (I, II, III). The calculation yielded Cohen's Kappa values of 0.71 and 0.87 for the ASD and TD groups, respectively.

#### Statistical Methods

Descriptive statistics were used to profile the scores of the questionnaires in each group. Continuous variables were summarized using means ±SD or median with interquartile range (IQR) and compared between groups using the Mann Whitney test. Categorical variables were summarized with counts and percentages. Agreement between raters was evaluated using Cohen's Kappa.

Repeated measures ANOVA was applied to evaluate the differences in communicative intentions over time and between groups and to evaluate the differences in the relative proportion of the communicative intentions.

*P*-values at 0.05 or below were considered significant.

Analyses were carried out using SPSS version 25.0, released 2017 (IBM Corp, Armonk, NY, USA).

## Results

### Chronological Age for Achieving the 40–70 Words Entry Criterion

As expected, the age by which the entry criterion was achieved was significantly higher among the toddlers with ASD (median = 28 months, IQR 24.5–35) in comparison to that of the toddlers with TD (median= 17 months, IQR= 17–18) (*p* < 0.001). Among the ASD group, the variability as expressed by the IQR was significantly larger (10-fold) as compared to the TD group (*p* < 0.001). Eighty-seven percentage of the toddlers with TD already possessed an expressive lexicon of 40–70 words by the age of 18 months, while none of the toddlers from the ASD group achieved this criterion before the chronological age of 20 months.

### Rate of Accumulating New Words

**Figure 1:**
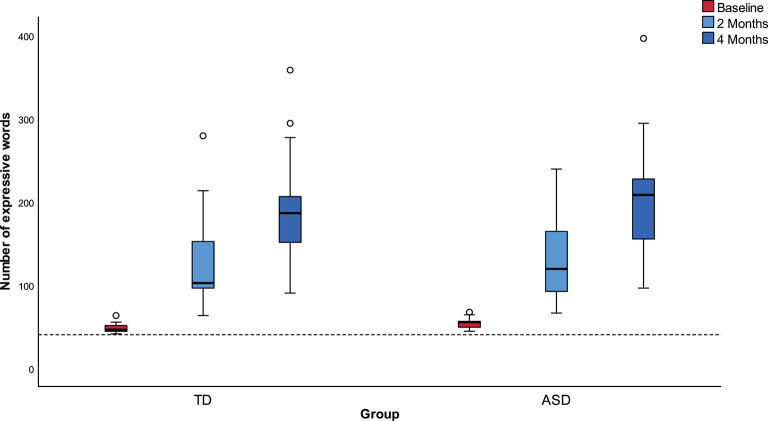
Mean numbers of expressive words at each visit in the two groups (TD and ASD).

The number of expressive words derived from the HCDI –WG is presented in Figure 1 for the two groups at each of the three visits.

Both groups significantly expanded the size of their expressive vocabulary (*p* < 0.001) and did so at a similar rate: an increase by 2.5 times from the first to the second visit and by 3.9 times on the third visit. There were no significant differences between groups regarding the course of expanding the vocabulary (*p* = 0.652). A high variation was found in both groups.

### Verbal Communicative Intentions' Trajectory

**Figure 2:**
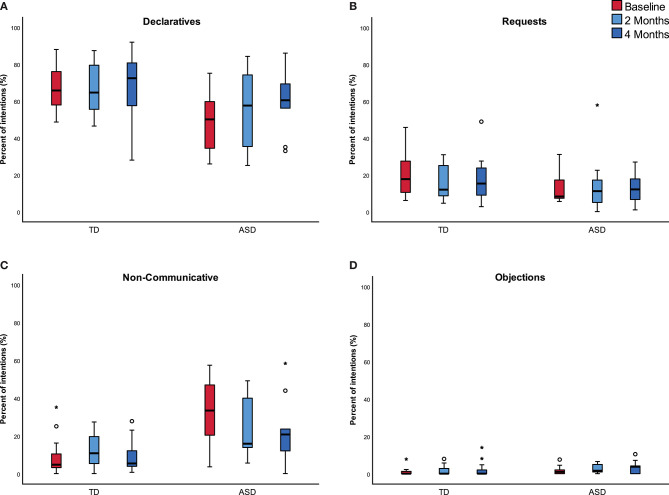
Proportion of communicative intentions in each group across three visits. In **(A)** declaratives, **(B)** requests, **(C)** non-communicative, and in **(D)** objections.

A total of 7,376 verbal productions was collected from the two groups over the 15 min sample of the three visits. As mentioned, each verbal production was exclusively classified into one of the five communicative categories. However, since the total number of verbal productions per 15 min sample increased from one visit to the next, we measured the proportion (%) of each category out of the total number of all verbal productions. Figure 2 presents the trajectory of those proportions (the category “others,” showing low values, was omitted from this figure).

To identify the contribution of the group and the visit on each communicative intention a repeated measure ANOVA model was applied yielding the following findings:

- Declaratives were the most prominent intention, significantly higher than all other communicative intentions (*p* < 0.05). This was the case for both groups and at all visits, with the exception of the first visit in the ASD group: while the proportion of Declaratives was higher than the Non-Communicative speech, this difference was insignificant (*p* = 0.224). The proportion of Declaratives remained similar during all three visits (*p*_time_ = 0.305). However, Declaratives were significantly higher among the TD group in comparison to the corresponding proportion in the ASD group across the three visits (*p*_group_ = 0.012).
- The proportion of Requests remained similar during all three visits with no significant differences between the groups (*p*_time_ = 0.820, *p*_group_ = 0.107).
- The proportion of Objections remained fairly low and stable during all three visits with no significant differences between the two groups (*p*_time_ = 0.181, *p*_group_ = 0.199).
- The proportion of the category of “others” remained similar during all three visits with no significant differences between the groups (*p*_time_ = 0.557, *p*_group_ = 0.942).
- Both groups used Non-Communicative speech. However, and as expected, the proportion of this category was significantly higher in the ASD group in comparison to the TD group (*p*_group_ = 0.001). Even though this category remained higher in ASD in comparison to TD across all three visits, we found an interaction effect (*p* = 0.012). In the ASD group, the Non-Communicatives decreased from 34.2 to 25.2% after 2 months and further decreased to 22.2% after 4 months, while in the TD group the proportion of this category remained stable and below 13% in all three visits.

## Discussion

The results of this study suggest both similarities and differences between toddlers with ASD and TD toddlers at similar lexical levels, regarding word onset, the rate of lexical growth, and the use of words for communicative purposes. As a group, toddlers with ASD reached the milestone of 40–70 words considerably later than TD toddlers. Moreover, the variability was higher in the ASD group as compared to the TD group, supporting previously-published research in other languages (1, 30). While toddlers with ASD were late in producing first words, we found that they accumulated new words at a similar pace to their TD peers. Smith et al. (31) reported that some participants with ASD demonstrated a rapid rate of vocabulary accumulation. Indeed, our results indicate that some toddlers with ASD, once passing an initial barrier, may proceed at a similar pace to their TD peers.

Our detailed analysis of the pragmatic intentions expressed via words revealed that Declaratives, rather than Requests, predominated the verbal productions of both groups. Participants in the two groups used their early words to comment on their surroundings or to name objects more often than to request them. Relatively speaking, the prevalence of Declaratives was higher in the TD group than in the ASD group.

Bearing in mind the difficulties toddlers with ASD have with cooperating in general and in sustaining interactions, we expected to identify a higher frequency of words expressing Objections, but this was not the case; low levels of words expressing Objections were found in both groups. Plumet and Veneziano (14) suggested that not the rate of oppositional episodes but rather the way they were handled distinguishes between children with ASD and language matched TD. A similar frequency of requests was found in both groups. This result is in accord with Paparella's findings (13) regarding similarities between toddlers with ASD and TD toddlers in the frequency of nonverbal requests.

The predominance of Declaratives and the low level of Objections may be the result of the naturalistic nature of the dyadic interactions during which our data was collected. Such style is thought to minimize potential conflicts and lower the need for request since items are available for the child's use. Therefore, it promotes sharing thoughts using Declaratives (32–34).

Alternatively, Requests and Objections, both serving imperative functions, are essentially different than Declaratives. To declare, attaching a label to objects will suffice. Thus, toddlers with ASD who are not necessarily impaired in mapping labels to objects may find the expression of this function relatively easy. However, aversive situations or needs may pose strong cognitive, emotional, and communicative demands, leaving toddlers with ASD unable to verbally express what they want or to ask for the termination of unwanted events. Thus, they may apply nonverbal means or withdraw from the interaction. Highly verbal children with autism have been described to have troubles using behavioral means including speech in diverse contexts (14). In addition, preference toward nonverbal means for resolving conflicts and achieving one's needs is actually rather common at early stages of typical language development, despite having adequate verbal means (35). In other words, the similar proportion of Requests between the two groups may be a byproduct of either the early stage or specifically the tendency of children with ASD to express Requests via nonverbal means. Further studies should explore whether preference toward nonverbal means in toddlers with ASD is restricted to early lexical stages or still characterizes later stages.

Perhaps the most interesting finding regarding the ASD group concerns their prevalent use of speech for non-communicative purposes, a phenomenon characteristic of this population (23). Uttering words with little communicative intention stands in sharp contrast to Objections, Requests, and Declaratives, which are addressed toward another person. As we expected, the proportion of Non- Communicative speech was greater in the ASD group than in the TD group throughout the three visits. While in TD toddlers the level of Non-Communicative speech remained low and steady, in our ASD group we noticed a gradual decline in this category as words accumulated. In other words, the number of productions addressed to the other in the ASD group rose from one visit to the next, though it did not quite reach the corresponding high level of communicative speech that TD toddlers achieved upon study entry.

The finding that TD toddlers use their speech mainly for communication comes as no surprise, as they convey communicative intentions via gestures and vocalizations for a prolonged period of time and so by the time they start uttering words, they are already proficient with the basic functions words may serve. Toddlers with ASD, on the other hand, arrive at a similar lexical point with limited pragmatic abilities and seem to hold on to a self-stimulatory function of speech. Only later, while expanding their active lexicon, do they gradually shift their speech to mainly use it for communicative purposes, though never completely abandoning the self-stimulatory function. Overall, the findings of the present study suggest that single word counts do not suffice when attempting to describe the linguistic profiles of toddlers with autism, as Dromi suggested previously (36). While their lexical acquisition could be described as a simple lag, when we examined the communicative functions of the words, a distinct pragmatic profile rose.

ASD toddlers' ability to develop an active lexicon and expand it is of great interest to language learning theorists. All toddlers face “the mapping mission,” trying to attach labels to objects and need to learn the extension range of different words in their native tongue (37, 38). The extent to which this process relies on social-pragmatic cues and whether grasping the pragmatic functions of speech is, indeed, the driving force of language development are still under debate (39). Past research has shown that toddlers with ASD tend to ignore social cues that signal the speaker's intentions while learning new words (7). Our findings add to this literature from the expressive perspective. Despite only having a partial understanding of what speech may be used for, toddlers with ASD were able to obtain an initial expressive lexicon. Such accomplishment may challenge the necessity of a fully intact pragmatic mechanism as a prerequisite to developing speech.

Our study's findings bear interesting clinical implications. First, the elevated levels of Declaratives may indirectly support the notion that the presence of specific pragmatic functions are highly influenced by contextual variables such as interaction style. Thus, whenever increasing the frequency of certain intentions is warranted, manipulating interaction from a directive to a facilitative style should be considered (32, 33).

Second, it should be emphasized that Requests and Objections enable the child to accomplish his needs and control his environment. Lack of appropriate verbal Requests and Objections is often associated with behavioral problems (40, 41). It is possible that some ASD toddlers substitute requesting and objecting by Non-Communicative means, hence the relative paucity of verbal requesting in our study. If further supported, it may highlight the need to teach appropriate requesting and objecting skills (36, 41).

Another clinical implication rises from the emerging ability of a naïve judge to accurately classify early word productions into the main pragmatic categories, with little training and achieving fairly good inter-rater reliability. It is possible that when the classification system is kept simple, referring to the broad categories of intentions, parents as well as other non-professionals may be able to attend to the pragmatic profiles instead of merely focusing on semantic development. With pragmatic deficits being one of autism's hallmarks, such ability seems promising.

## Limitation of the Study

The present study can be defined as a pilot study with relatively few participants with ASD. The standardization of our sample was based on their linguistic abilities rather than on their cognitive and severity scores. Future larger scale studies should control for differences in cognition and severity.

Two groups of participants with ASD were under-represented: minimally verbal ASD toddlers (not achieving our word criterion by 48 months) (42) and toddlers with ASD who develop an active lexicon on time (“Autism language normal”) (43). The latter group is often diagnosed at a later age, thus less available to research at such early stages. Further studies are warranted to explore whether those two groups display different early pragmatic profiles than the ones described in the present study.

In the current study we focused solely on verbal productions. Further studies call for analyzing verbal as well as nonverbal communication and the interaction between the two modes.

Since the pragmatic profile may be influenced by interaction variables, we suggest further studies addressing diverse elicitation techniques and contexts.

In the current study, toddlers with ASD were assessed as a group. Future studies may further define the association between an individual pragmatic profile and the rate of progress in expressive words.

## Conclusion

Our findings provide evidence that early lexical development among ASD toddlers, while delayed, shares both similarities and differences with respect to their pragmatic pattern as compared to TD toddlers. It is worth noting that lexicon may sometimes be achieved despite the lack of complete understanding as to the communicative nature of speech. Possibly among ASD toddlers, such insight is gained only later as their lexicon expands.

## Data Availability Statement

The raw data supporting the conclusions of this article will be made available by the authors, without undue reservation.

## Ethics Statement

The studies involving human participants were reviewed and approved by Loewenstein Rehabilitation Medical Center Institutional Review Board. Written informed consent to participate in this study was provided by the participants' legal guardian/next of kin.

## Author Contributions

AO and AM-B planned the study, performed it, analyzed it, and wrote it. ED planned the study, overviewed its performance, analyzed it, and wrote it. SG took part in performing the study, took part in its analysis, and reviewed the manuscript. All authors revised the final manuscript and approved it.

## Conflict of Interest

The authors declare that the research was conducted in the absence of any commercial or financial relationships that could be construed as a potential conflict of interest.
